# Attitudes, behaviours and barriers to public health measures for COVID-19: a survey to inform public health messaging

**DOI:** 10.1186/s12889-021-10790-0

**Published:** 2021-04-21

**Authors:** Raynell Lang, Jamie L. Benham, Omid Atabati, Aidan Hollis, Trevor Tombe, Blake Shaffer, Katharina Kovacs Burns, Gail MacKean, Tova Léveillé, Brandi McCormack, Hasan Sheikh, Madison M. Fullerton, Theresa Tang, Jean-Christophe Boucher, Cora Constantinescu, Mehdi Mourali, Braden J. Manns, Deborah A. Marshall, Jia Hu, Robert J. Oxoby

**Affiliations:** 1 Department of Medicine, Cumming School of Medicine, University of Calgary, Calgary, AB Canada; 2 Department of Community Health Sciences, Cumming School of Medicine, University of Calgary, Calgary, AB Canada; 3 Department of Economics, Faculty of Arts, University of Calgary, Calgary, AB Canada; 4 Primary Data Support, Data & Analytics, Alberta Health Services, Calgary, AB Canada; 5 School of Public Health, University of Alberta, Edmonton, AB Canada; 6 Department of Family and Community Medicine, University of Toronto, Toronto, ON Canada; 7 School of Public Policy and Department of Political Science, University of Calgary, Calgary, AB Canada; 8 Haskayne School of Business, University of Calgary, Calgary, AB Canada

**Keywords:** Coronavirus, COVID-19, Public health, Marketing, Behavior, Risk reduction, Attitudes, Compliance

## Abstract

**Background:**

Public support of public health measures including physical distancing, masking, staying home while sick, avoiding crowded indoor spaces and contact tracing/exposure notification applications remains critical for reducing spread of COVID-19. The aim of our work was to understand current behaviours and attitudes towards public health measures as well as barriers individuals face in following public health measures. We also sought to identify attitudes persons have regarding a COVID-19 vaccine and reasons why they may not accept a vaccine.

**Methods:**

A cross-sectional online survey was conducted in August 2020, in Alberta, Canada in persons 18 years and older. This survey evaluated current behaviours, barriers and attitudes towards public health measures and a COVID-19 vaccine. Cluster analysis was used to identify key patterns that summarize data variations among observations.

**Results:**

Of the 60 total respondents, the majority of persons were always or often physically distancing (73%), masking (65%) and staying home while sick (67%). Bars/pubs/lounges or nightclubs were visited rarely or never by 63% of respondents. Persons identified staying home while sick to provide the highest benefit (83%) in reducing spread of COVID-19. There were a large proportion of persons who had not downloaded or used a contact tracing/exposure notification app (77%) and who would not receive a COVID-19 vaccine when available (20%) or were unsure (12%). Reporting health authorities as most trusted sources of health information was associated with greater percentage of potential uptake of vaccine but not related to contact tracing app download and use. Individuals with lower concern of getting and spreading COVID-19 showed the least uptake of public health measures except for avoiding public places such as bars. Lower concern regarding COVID-19 was also associated with more negative responses to taking a potential COVID-19 vaccine.

**Conclusion:**

These results suggest informational frames and themes focusing on individual risks, highlighting concern for COVID-19 and targeting improving trust for health authorities may be most effective in increasing public health measures. With the ultimate goal of preventing spread of COVID-19, understanding persons’ attitudes towards both public health measures and a COVID-19 vaccine remains critical to addressing barriers and implementing targeted interventions and messaging to improve uptake.

**Supplementary Information:**

The online version contains supplementary material available at 10.1186/s12889-021-10790-0.

## Background

It has been one year since the World Health Organization (WHO) declared COVID-19 a global pandemic and many countries have endured multiple waves of disease during this time [1]. As of January 1, 2021, there have been over 100 million confirmed cases globally with over 2.2 million deaths [2]. The COVID-19 pandemic has resulted in unprecedented psychological, economic, and health implications, crippling healthcare infrastructure and placing cities under lockdown [3–6].

Support for public health measures including physical distancing, masking, staying home while sick, avoiding crowded indoor spaces and technology driven contact tracing (contact tracing and exposure notification applications) remain critical for reducing spread of the virus. Prior research has identified variability in individuals’ willingness to follow public health recommendations as well as accept a COVID-19 vaccine [7–10]. Demographic factors associated with greater uptake of public health behaviours include being female, older age (> 50 years old) and highly educated [9, 11]. Expressing higher concern for COVID-19 as well as greater knowledge of the pandemic is also associated with increased uptake of behaviours [9]. Using social media for COVID-19 information is associated with reduced adherence to public health recommendations and vaccine hesitancy, likely due to the widespread misinformation and conspiracy theories circulating on social media platforms [12–14].

Human behaviour is central to the spread of COVID-19 and therefore behavioural science must inform the public health response and communication strategies [15]. Persons make decisions by weighing the costs and benefits of choices; however, emotions often drive risk perceptions, possibly even more than information [16]. The media mostly uses negative framing for COVID-19, focusing on case counts or deaths, as opposed to recoveries. It is currently unknown which messaging themes, or if positive or negative frames, are most effective for increasing adoption of COVID-19 public health behaviours [16]. Michie et al. list several research areas needed to further the initial behavioural science research agenda for COVID-19 and the first two points include; 1) the need to evaluate population knowledge, anxiety, trust and attitudes towards public health measures, and the influence communication strategies have on these factors 2) the barriers and facilitators for public health interventions [15].

The aim of our work was therefore to understand current behaviours and attitudes towards public health measures, as well as barriers faced by individuals when trying to follow these measures. We also sought to identify concerns regarding COVID-19 vaccinations and reasons why persons may or may not accept a vaccine. We conducted a cross-sectional survey among Albertans targeting public health measures including wearing face masks in public spaces, physical distancing, staying home when sick, avoiding high risk indoor spaces, using contact tracing/exposure notification apps and willingness to get a COVID-19 vaccine when it is available. This work was the initial step of a multi-phase mixed-methods approach to inform data driven public health communication strategies, including knowledge translation tools, targeted marketing campaigns and community engagement to facilitate behaviour change.

## Methods

### Study design and population

A cross-sectional online survey was conducted in August 2020, in Alberta, Canada (Supplementary Material). The survey was created and designed by our research team and Alberta Health Services Primary Data Support team. A random sample of potential participants was contacted by phone using a purchased telephone sample of 3000 residential geocoded landline and cellphone numbers of Alberta residents. As this survey was the initial step of a multi-phase mixed-methods study, recruitment of participants was planned in order to subsequently conduct focus groups using the same participants [17]. Therefore, the sample size was based on the number of participants and parameters required for the focus groups. Participants were considered for inclusion if they were Alberta residents aged 18 years or older, spoke English and had internet access. Quotas were set to include participants for parameters including age and geographic region (Supplementary Material). Targeted geographic regions included; Calgary, Edmonton, other urban centers (i.e., Lethbridge, Red Deer, Medicine Hat), and rural (i.e., community or town not associated with or within 50 km of an identified urban center). Participation was voluntary and informed consent was obtained. The survey was distributed to each individual through email and conducted through Acuity, the online survey branch of Voxco [18]. All responses were aggregated and anonymized. All methods were carried out in accordance with relevant guidelines and regulations.

### Variables and measurement

The main outcomes were adoption of public health measures assessed by respondents answering the following question: Since the COVID-19 pandemic began, how often have you been performing each of the following: 1) physical distancing-trying to stay 2 m away from others, 2) wearing a mask in public, 3) downloading/using a contact tracing or exposure notification app 4) going to bars, pubs, nightclubs or lounges 4) staying home while even mildly sick. Adoption was assessed on a Likert scale of ‘all the time’, ‘most of the time’, ‘sometimes’, ‘rarely’ and ‘never’. A secondary outcome measure was willingness to receive a COVID-19 vaccine when available.

Sociodemographic factors were collected and categorized, including sex, region of residence, age, ethnicity, highest level of education, type/location of occupation, household income, usage of public transit, marital status and if children < 18 years old were living in the house. Likert scales were used to assess how effective persons believe public health recommendations are at reducing spread of COVID-19, how much these behaviours are influenced by the behaviours of others around them, how important they feel these behaviours are for preventing spread of COVID-19 and how difficult each of these behaviours are to perform. Usage of news sources and social media platforms were collected along with where persons get their health information about COVID-19. Of these sources of health information, participants were also asked to choose which ones were most trusted.

Participants’ experiences with COVID-19 were evaluated by asking if they or someone they knew had been tested for COVID-19 or have had COVID-19. Persons were asked if they had any medical conditions that may increase their risk for worse COVID-19 outcomes. Confidence in keeping themselves and their family safe from COVID-19 was also assessed. Likert scales were used to evaluate participants concern for getting or spreading COVID-19.

Awareness, downloads and usage of contact tracing/exposure notification apps were assessed. If persons had not downloaded an app, they were asked to provide specific reasons why. These responses were evaluated and aggregated into themes through an inductive approach. Participants were also asked if they would be willing to download an app if retail stores provided discounts to costumers with the app or if they would wear a mask if not wearing a mask resulted in a fine.

A series of risk and time preference questions were used to assess how persons valued risk and time trade-offs. Risk was assessed by asking persons their preference between*,* 1) a 30% chance of receiving $40 (with a 70% chance of receiving $0); or 2) receiving $20 for sure; this was asked using a series of different percentages. Time trade-offs were assessed by asking preference of receiving $40 in two weeks compared to $42 in six weeks; with a range of different values used for the six week interval (supplementary material: survey questions).

### Statistical analysis

Descriptive analysis was performed using crude data. All continuous variables had been categorized at the time of collection (Table 1). Demographic data were compared using the chi-square test. All *P*-values were two-tailed tests, and the statistical significance level set at *P* < 0.05. All statistical analyses were performed using STATA version 15.0 (College Station, TX).

**Table 1:** Demographic data

Characteristic		*N* = 60 (%)
Biologic Sex	Male	26 (43.3)
	Female	34 (56.7)
Geographic Location	Calgary	32 (53.3)
	Edmonton	15 (25.0)
	Other Urban centers*	7 (11.7)
	Rural	6 (10.0)
Age (years)	18–29	19 (31.7)
	30–59	26 (43.3)
	> 60	15 (25.0)
Ethnicity	White	51 (85.0)
	South Asian	3 (5.0)
	Chinese	2 (3.3)
	Filipino	1 (1.7)
	First Nation/Metis/Inuit	2 (3.3)
	Unknown	1 (1.7)
Highest Level of Education	High school diploma or less	10 (16.7)
	Post-secondary technical school	6 (10.0)
	Some college or University	12 (20.0)
	College or University diploma/degree	32 (53.3)
Occupation	Employed for wages	26 (43.3)
	Self employed	8 (13.3)
	Student	3 (5.0)
	Retired	8 (13.3)
	Not working	10 (16.7)
	Stay at home/maternity or paternity leave	5 (8.3)
Household Income (annual)	<$50,000	16 (26.7)
	$50,000–$100,000	15 (25.0)
	>$100,000	15 (25.0)
	Unknown	14 (23.3)
Work location during pandemic	Mostly going into work locations	18 (30.0)
	Mostly working from home	16 (26.7)
	Missing	34 (56.7)
Do you use Public Transit?	Almost Always	4 (6.7)
	Sometimes	4 (6.7)
	Rarely	19 (31.7)
	Never	33 (55.0)
Marital Status	Married/common law	37 (61.7)
	Separated/Divorced	6 (10.0)
	Widowed	1 (1.7)
	Never Married	16 (26.7)
Children < 18 yrs. living in house	Yes	19 (31.7)
	No	41 (68.3)

*Other urban centers included: Red Deer, Medicine Hat, Lethbridge and Grand Prairie

### Cluster analysis

Cluster analysis was used to identify key patterns that summarize data variations among observations. The goal was to estimate a limited number of clusters with the most similarity within clusters but most dissimilarity between clusters. Two practices of clustering analyses were performed based on two sets of variables in the data. The first analysis performed was clustering individuals based on news and social media consumption behaviours in general and for health related information, and the related trust questions about sources of news. The second cluster analysis was based on information about motivation factors that can affect individuals’ behaviour and their concern level in response to the pandemic. These factors came from the survey questions regarding experiences with COVID-19, concern and risk for COVID-19 and economic motivation questions.

We used Kmeans algorithm for clustering analyses to partition the dataset into four distinct non-overlapping clusters. This choice of algorithm and number of clusters were compared with other choices of methods (i.e., hierarchical clustering) and other numbers of clusters (i.e., two, three and five). Kmeans is an iterative algorithm that assigns observations or data points to a cluster with the objective to minimize the sum of squared distance between the data points and the cluster’s arithmetic mean of all the data points that belong to that cluster. The less variation seen within clusters, the more similar the data points are within the same cluster.

## Results

### Demographics

Of the 97 individuals who were emailed the online survey, 60 participants completed it. The majority of the surveyed population (*n* = 60) were female (34, 57%), from Calgary (32, 53%) and Whites (51, 85%) (Table 1). Those 18–29 years were more heavily sampled (19, 32%), 26 (43%) were ages 30–59 years and 15 (25%) were greater than 60 years old. The majority of persons were working (34, 57%) and educated with a college diploma or university degree (32, 53%). There were 9 (15%) persons who lived alone, 22 (37%) had children that lived with them, 9 (15%) lived with parents, 20 (33%) lived with other family members, 6 (10%) lived with non-family roommates and 4 (7%) listed other as their living situation.

There were 47 (78%) respondents who knew someone who had been tested for COVID-19 with 19 (32%) having been tested themselves. Thirteen (22%) knew someone who had COVID-19. There were 25 (42%) persons who reported having a medical condition that makes them more susceptible to COVID-19. The level of concern for getting COVID-19 was overall less than the level of concern for spreading COVID-19. Only 15% of persons reported they were extremely concerned about getting COVID-19 whereas 15% of persons expressed they were not at all concerned about spreading COVID-19. When asked if they felt confident that they could keep themselves and their family safe from COVID-19, 38 (63%) agreed.

### Current behaviours

#### Uptake of public health measures

**Fig. 1:**
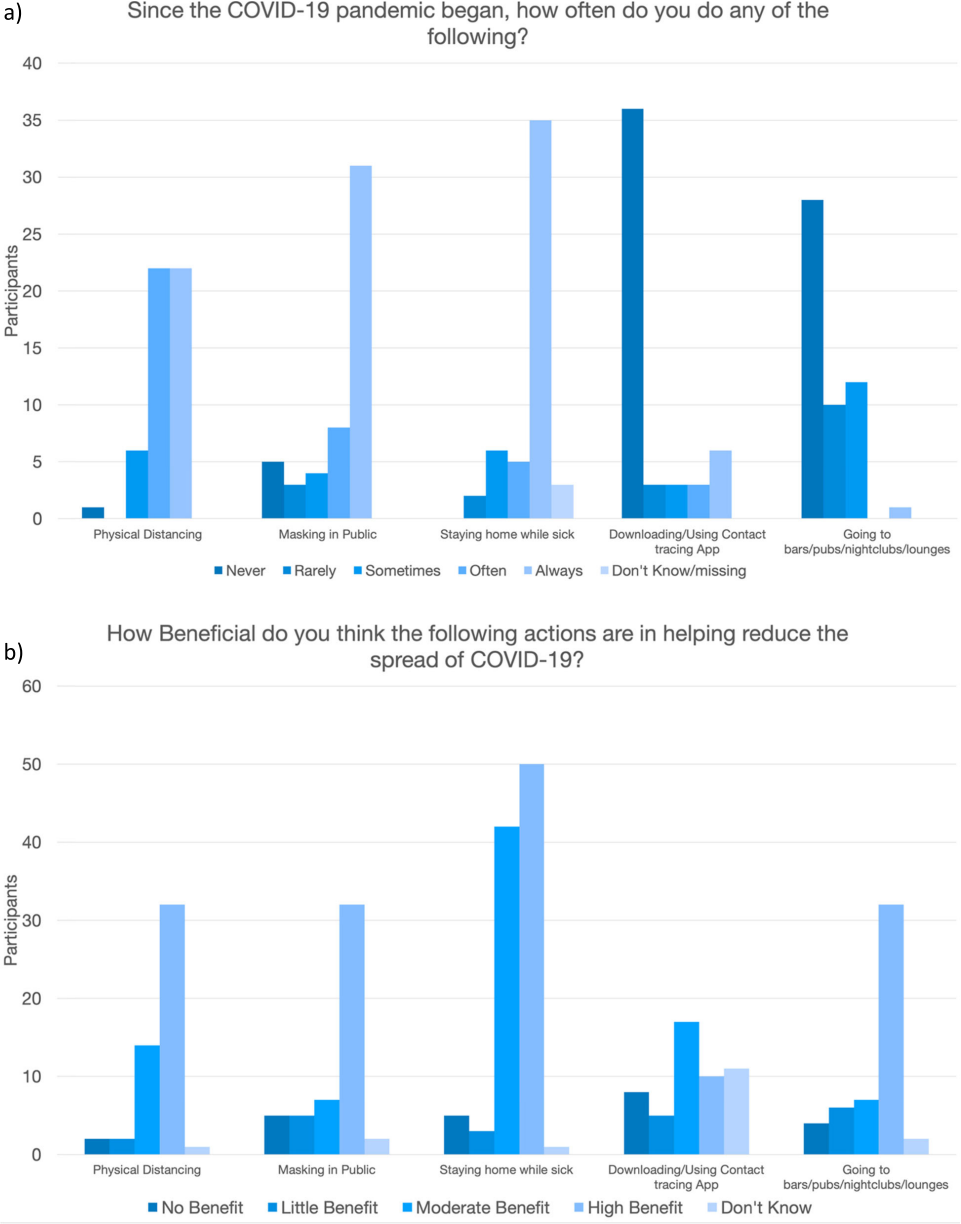
**a**. Compliance with Public Health Behaviours. (*n* = 60). **b**. Perceived benefits of Public Health Behaviours. (*n* = 60)

The public health measure that was reported by most as always followed was staying home while sick (43, 73%) (Fig. 1a). Staying home while sick was also perceived by most as having a high benefit (51, 86%) for reducing the spread of disease (Fig. 1b). The majority of persons reported that they always wear a mask in public (36, 60%) and either always (26, 43%) or often (27, 45%) physically distance. There were 28 (47%) respondents who said they never go to bars/pubs/nightclubs or lounges.

#### Uptake of a contact tracing/exposure notification app

There were 41 (68%) people who were aware of the COVID Alert exposure notification app and 47 (78%) aware of the “ABTraceTogether” contact tracing app for COVID-19 and 14/49 (29%) people had downloaded it. Privacy concerns and lack of knowledge on app logistics were the most common barriers listed to app use; however, other themes included not feeling an app was needed and digital barriers (i.e., not having a cell phone, not using apps). Of the 14 persons who downloaded the app, 9 (64%) thought it should be required. However, of the 34 persons who did not download the app, 22 (65%) thought it should not be required. Of those who did not download the app, 12 (35%) said they would download it if they received grocery store discounts as a result. Among persons who felt confident that they could keep themselves and their family safe from COVID-19, only 6 out of 30 (20%) downloaded the app. App downloads did not vary by sex, age, ethnicity, education, income, occupation or rural vs urban locations. All persons who had reported they had no concern for getting or spreading COVID-19 had not downloaded the app; however, 38% of persons who had extreme concern about getting and 31% of persons with extreme concern of spreading disease had downloaded the app.

#### COVID-19 vaccine acceptability

There were 41 (68%) persons who would receive a COVID-19 vaccine if it were available, with 12 (20%) saying they would not and 7 (12%) being unsure. This was greater than the proportion who had received the annual influenza vaccine since 2018 (47%). When divided by ethnicity, 32 (63%) White participants would be willing to receive a vaccine, compared to 100% of persons of other ethnicities. Those with higher education levels (college or university attendance) were less likely to receive a COVID-19 vaccine if it were available (63%), compared to 100% of those who attended post-secondary technical school or 70% of those with a high school diploma or less education. Sixteen percent of persons who had attended university or college were uncertain if they would accept a vaccine. Willingness to get a COVID-19 vaccine was less in other urban centers (29%) and rural Alberta (50%) compared to Calgary (75%) and Edmonton (80%), (*P* = 0.030). Of the nine participants who reported living with their parents, all would be willing to receive a vaccine, compared to 63% of those who did not live with parents (*P* = 0.086). Vaccine uptake was not associated with age, sex, income or occupation.

All persons who reported they were either extremely concerned or very concerned about getting COVID-19 reported that they would take a COVID-19 vaccine. Whereas only 20% of those who had no concern for getting COVID-19 would receive a COVID-19 vaccine (*P* = < 0.001). Of those with extreme concern for spreading the virus, 100% said they would receive a vaccine, compared to 44% who had no concern for spreading the virus (*P* = 0.006). Persons who reported they would accept a COVID-19 vaccine were also more likely to be compliant with public health measures including staying home when sick (*P* = 0.033), masking in public (*P* < 0.001) and physical distancing (*P* = 0.005).

### Barriers and attitudes towards following public health measures

#### Belief in the efficiency of public health measures

The majority (51, 86%) of persons felt that staying home while even mildly sick would have high benefit to reducing spread of COVID-19. Downloading and use of a contact tracing/exposure notification app was the least adopted public health measure with 60% of persons reporting they had never used it and only 10% reporting always using it. A contact tracing/exposure notification app was also believed to be least beneficial, with 15% of persons believing it to be of no use in reducing transmission, 13% feeling it had little benefit and 30% reporting moderate benefit (Fig. 1b).

#### Influence of others on participants compliance with public health measures

**Fig. 2:**
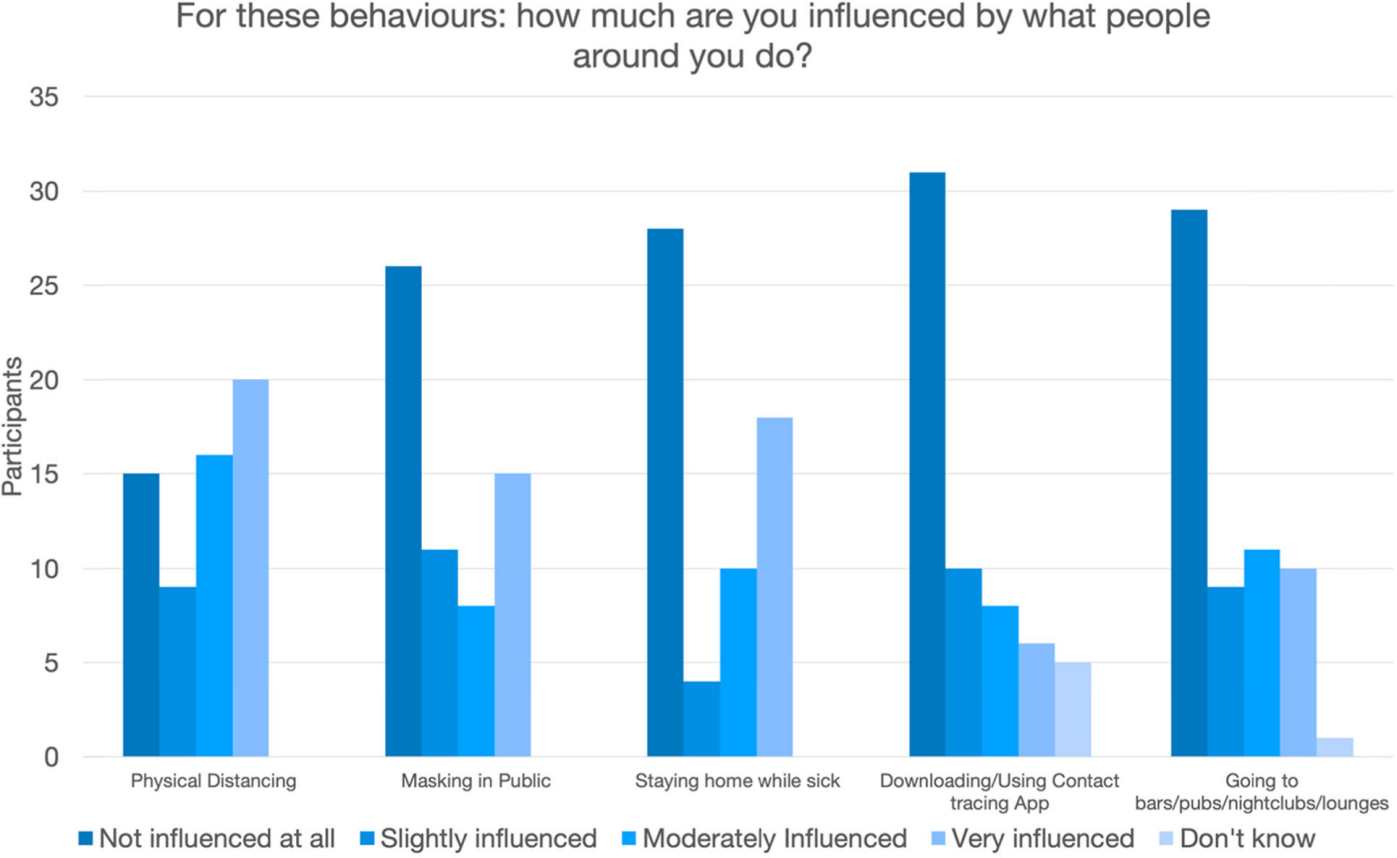
Influence from others on your compliance with these public health measures. (*n* = 60)

Persons reported they were very influenced to physically distance if others were doing the same (20, 33%). Other measures most influenced by others were staying home while sick (18, 30%) followed by masking in public (15, 25%). The majority of persons (31, 52%) reported that they were not influenced at all by others having a contact tracing/exposure notification app (Fig. 2). There was no association identified between age, sex, income, occupation, education or ethnicity on whether persons were influenced by others on any of the evaluated public health measures; however, persons living in Calgary and Edmonton felt more influenced to physically distance if persons around them were doing so (*P* = 0.014), compared to persons living in other urban or rural centers.

#### Importance vs difficulty of performing public health measures

**Fig. 3:**
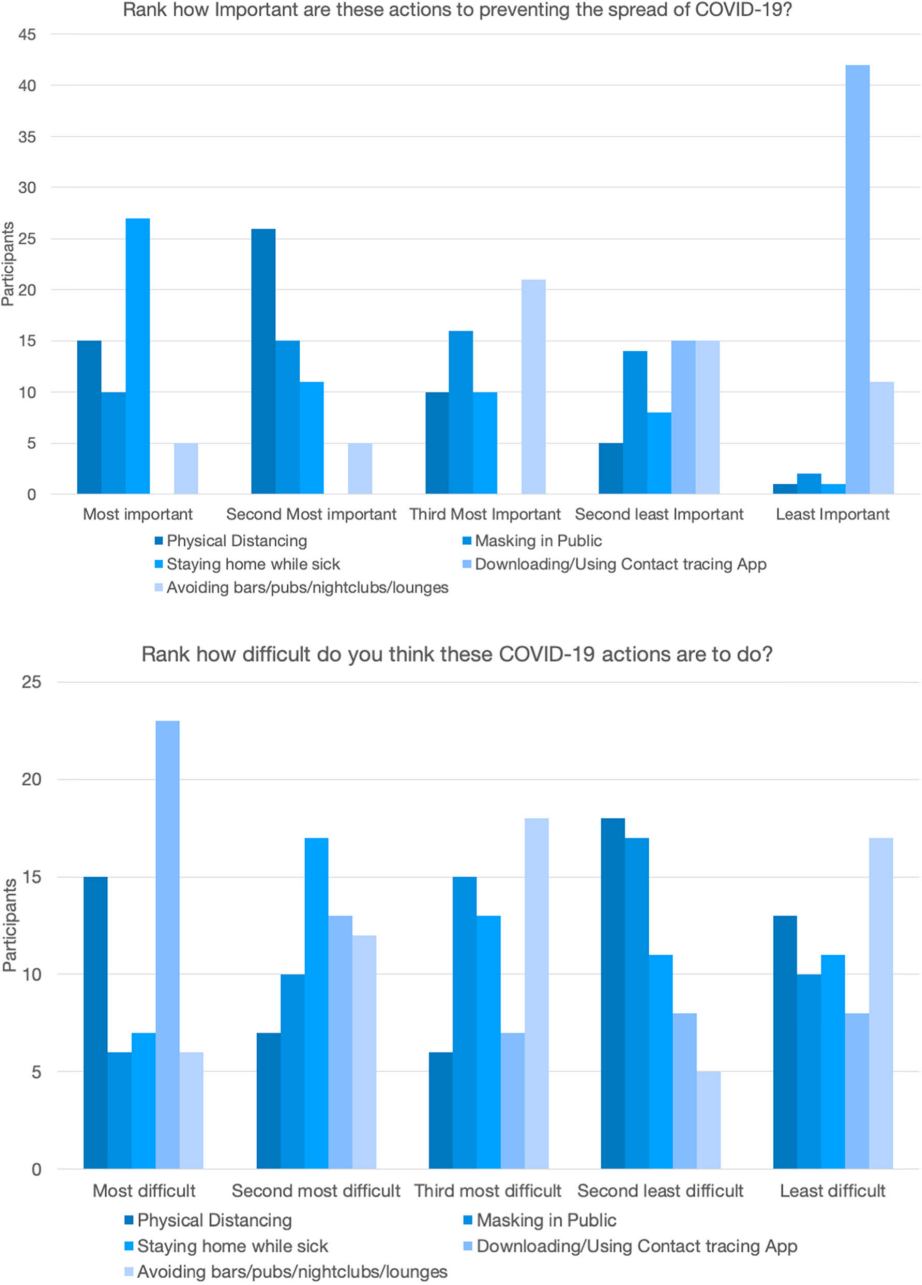
**a** Ranked importance in prevention of COVID-19 spread for each public health measures. (*n* = 60). **b**. Ranked difficulty in performing each public health behaviour. (*n* = 59)

When asked to rank which measure was most important in preventing spread of COVID-19, 47% reported staying home while sick, followed by 26% saying physical distancing, 18% masking in public, and 9% avoiding bars/pubs/nightclubs and lounges (Fig. 3a). Of all measures, 74% of persons felt that a contact tracing/exposure notification app is least important in preventing spread of disease. Persons also felt that downloading/using an app would be most difficult to do (40%) (Fig. 3b). The greatest number of persons reported avoiding bars/pubs/nightclubs and lounges as the least difficult to do (29%), followed by physical distancing (22%).

Persons in the youngest age group found it least difficult to mask in public, whereas the middle and oldest age group felt it was least difficult to avoid bars, pubs, nightclubs or lounges (*P* = 0.041). Persons living in Calgary and Edmonton were more likely to report physical distancing, staying home while sick and avoiding bars/pubs/nightclubs or lounges as most difficult compared to persons living in other urban or rural centers who reported masking in public as more difficult (*P* = 0.004). Otherwise, there were no associations noted between reported difficulty of these measures with sex, age, income, occupation, education or ethnicity.

### Public health communication platforms

#### Platforms for information used and trusted by participants

**Fig. 4:**
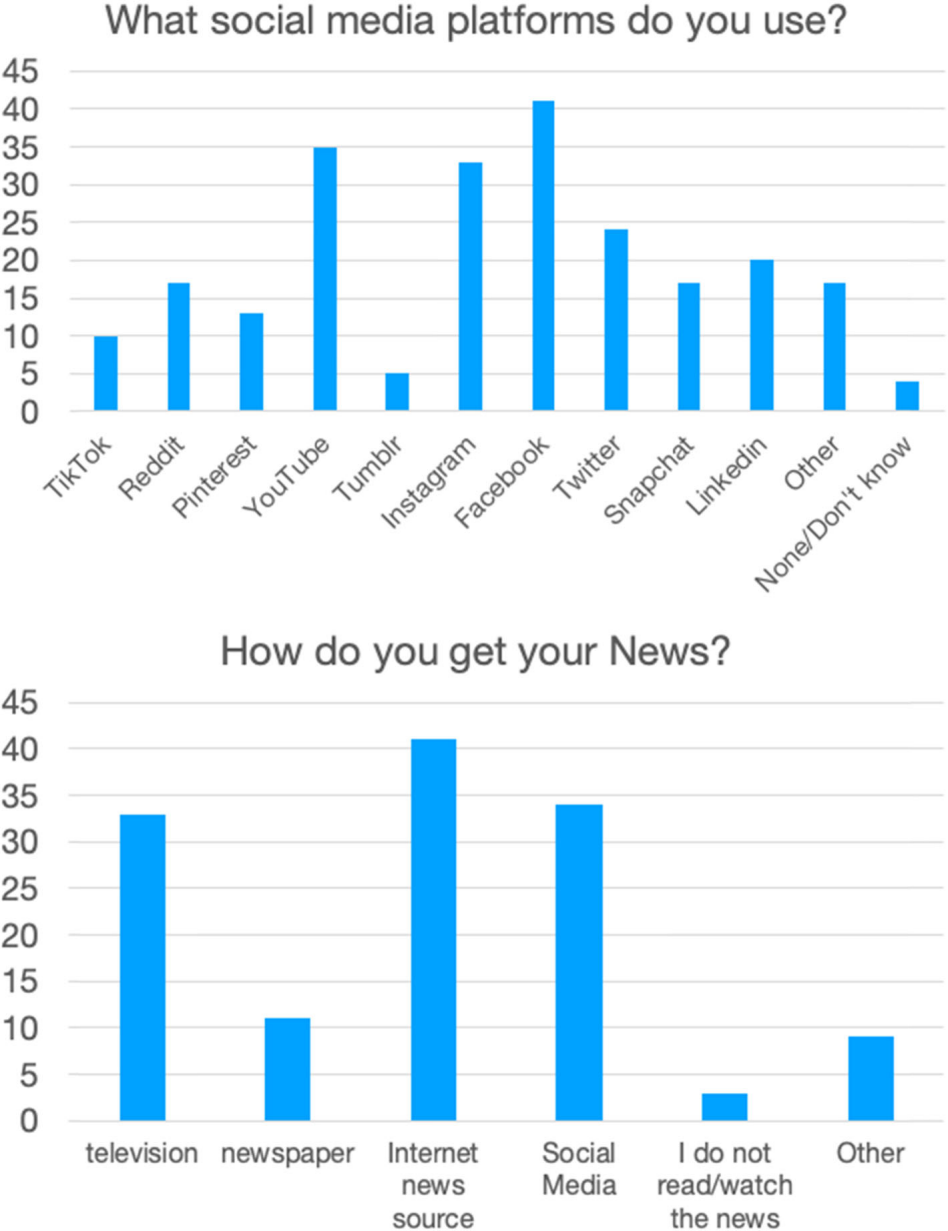
Platforms for social media and news. (*n* = 60)

The majority of persons used Facebook™ (68%), YouTube™ (58%) and Instagram™ (55%). Only 7% of respondents said that they used no social media or did not know. The majority of persons received their news from internet news sources (68%), however 57% said they received their news on social media (Fig. 4).

**Fig. 5:**
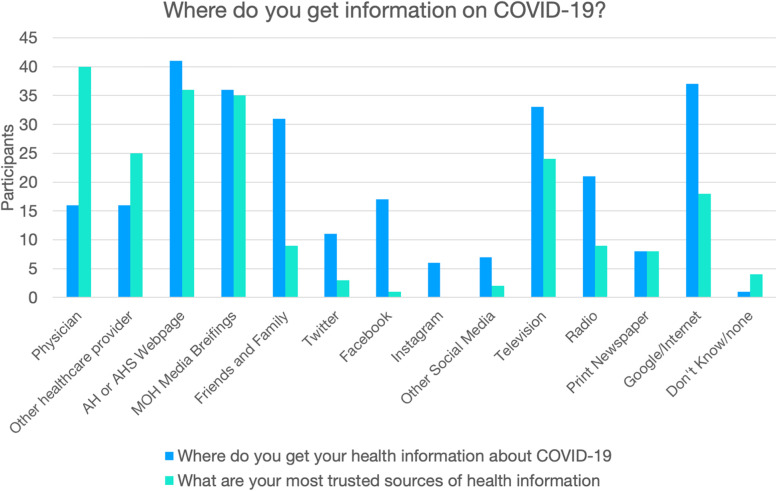
Platforms for health information. (*n* = 60). (AH) Alberta Health, (AHS) Alberta Health Services, (MOH) Chief Medical Officer of Health

The majority of persons get their COVID-19 health information from Alberta Health or Alberta Health Services webpages (68%). These webpages are also trusted with 60% listing them as one of their most trusted sources of health information. The second most common source of health information was google/internet (62%), however this source was much less trusted with only 30% listing it as a trusted source. Chief Medical Officer of Health (MOH) media briefings were also highly utilized (60%), and trusted (58%). Health information from physicians was most trusted, with 67% of participants listing it as one of their most trusted sources of health information; however, not well utilized with only 27% reporting that they obtain their COVID-19 information from a physician. (Fig. 5). Those who received their health information from the MOH media briefings (*P* = 0.030) and Alberta Health or Alberta Health Services websites (*P* = 0.040) were significantly more likely to accept a COVID-19 vaccine. Those who received their health information from Facebook were less likely to receive a COVID-19 vaccine (47%) (*P* = 0.08).

### Cluster analysis to archetype Behaviours by attitudes, barriers and information sources

In cluster analysis 1 (Table 2), the “summary of description” identified four clusters based on news and social media consumption and the relevant trust related questions. Cluster analysis 1 aimed to understand whether we could identify correlations and patterns in the data based on news and social media consumption behaviors of individuals. There were three key insights from cluster analysis 1. First, clustering distinguished between individuals who used more traditional news sources (i.e., TV) versus those who used social media sources for general and/or health related issues. Second, clustering showed social media use as a news source was correlated with individuals’ reported tendency to be influenced by others in their health related behaviors. Third, the reported trust level in health information on COVID-19 from health authorities was a key factor in distinguishing clusters.

**Table 2:** Clustering Analysis 1-Clustering based on social media, news source, trust questions

Clusters	1	2	3	4
**Summary of description (on average)**	**-Low social media use.** **-Use TV for news** **-Have trust in health authority.** **-Low effect of social influence on public health behaviour choices**	**-Use social media also use it for news.** **-Lower trust on health authorities** **-Lower effect of social influence except distancing and staying home while sick**	**-Use social media but not for news.** **-Lower trust on health authorities** **-Good effect of social influence except on use of app**	**-Use social media also for news** **-Have trust in health authorities** **-Higher effect of social influence**
Downloaded an app (%)	0	12	11	24
Accept a vaccine (%)	30	15	25	50
Age				
1: < 30	4.78	2.29	2.57	2.26
5: + 60				
Reason for no download: Privacy concern in app (%)	35	23	7	20
Reason for no download: No need in app (%)	35	11	7	13
Reason for no download: Lack of awareness (%)	21	35	57	26
Risk and Time preference	More likely to accept a lower risk/sooner payoff	Medium level of risk and time preference	Medium level of risk and time preference	More likely to accept a higher risk/later payoff
How often do you:				
1:Rare, 3:Always				
Physical distance	2.9	2.8	2.8	2.9
Use a mask	2.9	2.3	2.5	2.8
Stay home if sick	3	2.9	2.5	2.9
Use contact tracing/exposure notification app	1	1.5	1.6	1.7
Avoid bars, clubs, nightclubs and lounges	1.35	1.3	1	1.3
Confident keeping you/family safe: Yes = 1. No = 2	1.57	1.4	1.35	1.8
Concern of getting COVID-19 1 very, 4: none	2.35	3	3.3	2.46
Observation count	14	17	14	15

Below are the means across clusters developed from the data (four clusters, minimizing distance)

Other correlation patterns identified included a greater use of the app and uptake of potential vaccines for COVID-19 associated with greater level of social media use. Reporting health authorities as most trusted sources of health information in Clusters 1 and 4 was associated with greater percentage of potential uptake of vaccine, but not related to use and download of an app. Cluster 1 included the group of individuals with lower use of social media, more use of traditional news sources that consisted of older individuals, who had not downloaded the tracing app and expressed greater privacy concerns as a barrier to use. Cluster 1 reported a tendency to accept a lower risk payoff, and a payoff closer to present as opposed to a larger payoff in a future time. The largest difference in public health measures between clusters were identified in the usage of the tracing app and uptake of a potential COVID-19 vaccine. There was less dissimilarity between clusters in use of a mask, avoiding public places, staying home if sick, and physical distancing.

In cluster analysis 2 (Table 3), the “summary of description” describes the basis of creation of the four clusters. Cluster analysis 2 aimed to understand whether we could identify meaningful patterns in the health and economic motivators, concerns and health related information. This analysis highlighted four key points. First, the level of concern of getting and spreading COVID-19 was fundamental in creating clusters. Second, in persons who expressed the least concern for COVID-19 (Cluster 1), introducing a fine was partially positive in persuading the use of masks and was not effective for other clusters. Third, a subgroup of individuals with lower concerns in getting and spreading COVID-19 (Cluster 1) were the group with least impact from an offer of discount for use of a contact tracing/exposure notification app. This cluster also contained the most negative responses to requiring customers to use masks and/or apps in businesses and had the lowest percentage of persons having had an influenza vaccine since 2018. Fourth, comparing Clusters 2 and 4 showed that the group who were less concerned about getting but more concerned on spreading COVID-19 (Cluster 2) were more confident in keeping themselves safe from COVID-19, more likely to work from home, and had a negative reaction to having business requiring their customers to use mask or the tracing app. Both Clusters 2 and 4 provided partially positive responses for the effect of offering discounts for use of the tracing app.

**Table 3:** Clustering Analysis 2- Clustering based on health and economic motivators

Cluster	1	2	3	4
**Summary of description**	**-Lower concern in getting and spreading COVID-19** **-Unsure/mixed reaction to a fine for not wearing mask (use masks less often than cluster 3)** **-No influence from discount** **-No Flu shot** **-More negative reaction to business requiring masks or use of app**	**-More concerned to spread COVID-19** **-Wear mask regardless of fine** **-Discount works positively for use of app** **-Some had flu shot** **-Negative view in business requiring use of app** **-More likely to work from home than cluster 4**	**-Lower concern in getting and spreading COVID-19** **-Fine is ineffective, as they wear mask regardless of fine** **-Discount works positively for use of app** **-Some had flu shot** **-Less negative reaction to business requiring masks or use of app**	**-Very concerned** **-Wear mask regardless of fine** **-Discount works positively for use of app** **-Some had flu shot** **-Mixed/ less negative view of business requiring use of app** **-Less confidence in keeping themselves safe than cluster 2**
Concern in getting COVID-19 (average) 1:high/4: low	3.8	2.8	3.3	1.3
Concern in spreading COVID-19 (average) 1:high/4:low	3.3	1.7	3.3	1.1
Had Flu shot	0	6	8	10
Do not feel confident in keeping safe from COVID-19(%)	Low	Low	Low	35
Effectiveness of Fine for use of mask	1 Wears mask regardless, 2 Would with fine, 4 Would not, 4 Not sure	100% Wear mask regardless	11 Wear mask regardless, 2 Not sure	85% Wear mask regardless
Discount offer to download app works?	No	12 Yes, 7 Don’t know	7 Yes, 3 Not sure	11 Yes
Work from home (%)	50	40	40	5
Have high risk health conditions	6	6	2	11
Tested for COVID-19	2	9	5	2
Know someone tested for COVID-19	8	20	10	8
Know someone who had COVID-19	2	6	3	2
Use of social media (%)	70% social media for health news	30% social media for health news	30% social media for health news	70% social media for health news
Have not downloaded an app (%)	95	80	100	80
Willingness to take a COVID-19 vaccine	8 No, 3 Not sure	6 Yes, 15 No	2 Yes, 9 No, 2 Not sure	11 Yes, 3 No
Observation counts	11	21	13	14
How often do you: 1:Never/ 4: Always				
Physical distance	2.5	2.9	2.9	2.9
Use a mask	1.5	2.9	2.8	3
Stay home if sick	2.6	3	2.8	2.9
Use contact tracing/exposure notification app	1.4	1.5	1.2	1.7
Avoid bars, clubs, nightclubs and lounges	1.5	1.3	1.2	1

Below are the means across clusters developed from the data (four clusters, minimizing distance). (Numbers are observation counts if not stated as % or not given a rubric)

By projecting cluster indicators to other information in the data, Table 3 highlighted that (i): lower concerns of getting and spreading COVID-19 (Clusters 1 and 3) showed the least uptake of public health behaviors except for avoiding public places such as bars, (ii): lower concerns regarding COVID-19 is associated with more negative responses in taking a potential COVID-19 vaccine, (iii): the group with lower concern of getting and more concern of spreading COVID-19 showed lower tendency to take a potential vaccine.

## Discussion

With this work we describe current behaviors of wearing face masks in public spaces, physical distancing, staying home when sick, avoiding high-risk spaces like crowded indoor gatherings, using contact tracing apps and willingness to take a vaccine when available. We demonstrated that the public health measure most followed was staying home while sick, which was also perceived by most as having a high benefit to reducing the spread of disease. Downloading and using a contact tracing/exposure notification app was performed least, with the perceived lowest benefit to reducing spread of COVID-19 and also reported as most difficult to do. Least difficult was avoiding bars/pubs/nightclubs or lounges, however this did vary by age group. Persons living outside of Edmonton/Calgary felt masking in public was the most difficult measure to perform.

The need to understand behaviour and how to change it through applying behavioural science methods and models was recently highlighted by West et al. [19]. Their paper emphasizes an urgent need for effective interventions to increase adherence to public health measures [19]. The authors recommend using the capability, opportunity, motivation and behaviour (COM-B) model to determine current behaviours to inform a data-driven intervention [19, 20]. The capability to adopt public health measures requires people to understand what needs to be done, how and why [19]. Believing that public health behaviours are effective is as an important predictor of compliance with these behaviours [21]. This is likely why staying home while sick is the most practiced behaviour, as it is also believed by respondents to be the most important in preventing COVID-19 transmission.

Unfortunately, few people felt that a contact tracing/exposure notification app would be of high benefit in reducing the spread of COVID-19 and therefore likely explains its poor uptake. Rapid and accurate contact tracing remains a pillar to the COVID-19 response. Mathematical models found that prompt contact tracing and isolation of exposed persons combined with a large scale testing program could effectively control the pandemic [22–24]; however, app success is dependent on broad uptake and utilization. Early models of COVID-19 transmission demonstrated that approximately 60% population uptake of these apps is needed to slow disease transmission [25]; however, recent models suggest that these apps may still be effective at much lower levels of adoption [26–28]. Extensive messaging and education on app logistics and privacy concerns are needed to improve uptake to a level where the contact tracing app would be of significant benefit.

The willingness for COVID-19 vaccination in this population was much greater (68%) than in other Canadian surveys that reported only 39% of Canadians would get a COVID-19 vaccine as soon as it was available [29]. The differences seen in willingness for vaccine uptake between our sample and other Canadian surveys may reflect the method of sampling that was used in our study. However, vaccine hesitancy surrounding COVID-19 has the ability to thwart control of the pandemic [30]. It is important to understand the attitudes and barriers to vaccine uptake to promote behaviour changes in those unwilling to receive a vaccine. Persons with college or university education expressed reduced willingness to receive a vaccine as did those living in smaller urban or rural locations. Persons with greater compliance for other public health interventions were more likely to accept a vaccine as were those that expressed greatest concern for either getting or spreading COVID-19. Segmented communication towards persons with factors associated with lower willingness to receive a vaccine including persons living in small urban or rural locations, Whites, highly educated persons and persons reporting little concern for COVID-19 should be developed.

The majority of respondents used some form of social media with the top three being Facebook, YouTube or Instagram. Persons using social media for health information are less likely to accept a COVID-19 vaccine. Attitudes towards immunization are shaped by the information and ideas that individuals are exposed to online, and in particular, through social media [31–33]. Social media should be utilized as a platform to disseminate public health communications promoting protective behaviours.

The cluster analysis highlights clear patterns in the data, identifying clusters of persons with similar attitudes and behaviours and the association with demographics, facilitators and barriers to public health behaviours. From this information, archetypes of particular characteristics associated with non-adoption of public health behaviours were analyzed to provide information for segmented communication and tailored messaging towards these groups of individuals to promote behaviour change. Individuals’ concern level regarding getting and spreading COVID-19 have a significant association with protective health behaviors including use of masks, social distancing and use of an app. Marketing campaigns that increase concern for COVID-19 may be effective in increasing uptake of public health behaviours; however, this will require a fine balance as to not promote fear appeal, which is a controversial method of promoting behaviour change [16, 34, 35]. Fear appeals have been demonstrated to produce the greatest behaviour changes when persons feel capable of managing the threat, however may otherwise lead to defensive responses [16].

Individuals who expressed greater social influence can be persuaded to public behaviors and generally show more protective health behaviours. However, a more individual persuasion is needed for persons with lower reported social influence and use of social media. Persons of different ages and regions of residence may respond to different messaging themes and benefit from segmented communication. Younger respondents reported greater difficulty with not going to bars, pubs, nightclubs or lounges, whereas older respondents felt masking in public to be most challenging, therefore messaging should be segmented by age and tailored towards the public health behaviour reported most difficult by that age group. Persons living in more rural centers found it more difficult to mask in public, whereas persons in more urban regions reported physical distancing as more difficult and were also influenced by persons around them physically distancing. Therefore, segmented and tailored messaging can be used for persons of rural and urban regions taking into account these differences. Regarding the economic motivators, introducing fines for not using masks were not effective, but there are some partial positive responses for offering discounts for use of the tracing app, even among those with lower health concerns.

There are several study limitations that should be noted. We did have small numbers of participants and therefore generalizability may be limited. Through the use of a quota system we were able to gather data on persons of different ages, sex, and within different locations of our province; however, this was developed for the subsequent focus group composition and therefore may not be representative of all persons in Alberta. The result of cluster analyses should be evaluated with caution as the sample size is small particularly when data points are subdivided. Our recruitment strategy involved use of a random sample of phone numbers from across Alberta. Individuals with more than one number (e.g., a landline and a mobile phone number) could have had an increased chance of selection if both numbers were included in the random sample. Due to the small sample size, our comparative analysis may not have reached statistical significance despite a difference seen.

## Conclusions

The majority of persons indicated that they were physically distancing, masking and staying home while sick either always or often. There was variation noted with age groups, with persons in the youngest age group finding it least difficult to mask in public, whereas the middle and oldest age groups felt it was least difficult to avoid bars, pubs, nightclubs or lounges. A large proportion of persons were identified who have not downloaded a contact tracing/exposure notification app and who would not receive a COVID-19 vaccine when available. Reporting health authorities as most trusted sources of health information was associated with greater percentage of potential uptake of vaccines. Persons with lower concern for COVID-19 demonstrated the least uptake of public health measures and a COVID-19 vaccine. These results suggest informational frames and themes focusing on individual risks, highlighting concern for COVID-19 and targeting improving trust for health authorities may be most effective in increasing public health measures.

This work significantly contributes to the COVID-19 literature through an in-depth characterization of attitudes and barriers towards public health behaviours, contact tracing/exposure notification apps and COVID-19 vaccines. Findings from this study can inform future COVID-19 research and provide useful information for the development and implementation of targeted interventions and messaging to improve uptake of public health behaviours.

## Supplementary Information

**Additional file 1: Supplementary Material.** Survey Questions

## Data Availability

The summary dataset used and or analyzed during the current study are available from the corresponding author on a reasonable request.

## Abbreviations

WHO: World Health Organization
AH: Alberta Health
AHS: Alberta Health Services
MOH: Minister of Health
