# Supplementary material for: Attitudes, behaviours and barriers to public health measures for COVID-19: a survey to inform public health messaging
Source: BMC Public Health. 2021 Apr 21;21:765. doi: 10.1186/s12889-021-10790-0 (PMC8058588; doi:10.1186/s12889-021-10790-0)
Supplement: Supplementary file 1 — Additional file 1: Supplementary Material. Survey Questions [file 12889_2021_10790_MOESM1_ESM.docx]

**Attitudes, Behaviours and Barriers to Public Health Measures For COVID-19: A Survey to Inform Public Health Messaging**

Raynell Lang MD^1^, Jamie L. Benham MD^1,2^, Omid Atabati MSc^3^, Aidan Hollis PhD^3^, Trevor Tombe PhD^3^, Blake Shaffer PhD^3^, Katharina Kovacs Burns MSc MHSA PhD^4,5^, Gail MacKean PhD^2^, Tova Léveillé MA^4^, Brandi McCormack MSc^4^, Hasan Sheikh MD MPA^6^, Madison M. Fullerton MSc^2^, Theresa Tang BHSc(Hons)^2^, Jean-Christophe Boucher PhD^7^, Cora Constantinescu MD MSc^1,2^, Mehdi Mourali PhD^8^,  Braden J. Manns MD MSc^1,2^, Deborah A. Marshall PhD^1,2^, Jia Hu MD^2^, Robert J. Oxoby PhD^3^

^1^ Department of Medicine, Cumming School of Medicine, University of Calgary, Calgary, AB, Canada

^2^ Department of Community Health Sciences, Cumming School of Medicine, University of Calgary, Calgary, AB, Canada

^3^ Department of Economics, Faculty of Arts, University of Calgary, Calgary, AB, Canada

^4^ Primary Data Support, Data & Analytics, Alberta Health Services, AB, Canada

^5^ School of Public Health, University of Alberta, Edmonton, AB, Canada

^6^ Department of Family and Community Medicine, University of Toronto, Toronto, ON, Canada

^7^ School of Public Policy and Department of Political Science, University of Calgary, Calgary, AB, Canada

^8^ Haskayne School of Business, University of Calgary, Calgary, AB, Canada

Corresponding Author: Jia Hu, jia.hu@albertahealthservices.ca

**Supplementary Material: Survey Questions**

*The following questions ask your knowledge, beliefs and use of contact tracing apps.*

- **Before this survey, were you aware of the Alberta COVID tracing app, ABTraceTogether?** [Y/N]
- **Before this survey, were you aware of the Federal COVID tracing app, COVID Alert?** [Y/N]
- **Have you downloaded either of these contact tracing apps?** [Y/N]
- **If no, then why?** [Free text]
- **If you have downloaded the app, do you use it?** [Y/N]
- **If no, then why?** [Free text]
- **Based on what you know about these tracing apps, do you think businesses should require employees and/or customers to download and use the app?** [Y/N]
- **If a business required the use of a contact tracing app to enter, would you view this as protecting you?** [Y/N]
- **Would you be willing to download the app if a grocery store (or other retailer) offered discounts for customers with the app?** [Y/N]
- **If yes, how large of a discount would the grocery store (or other retailer) need to offer for you to be willing to download the app? (If no, skip)** 5%, 10%, 15%, 20%, 25%, >25%

*The questions below help are about your knowledge, beliefs and practices around public health actions.*

Since the COVID-19 pandemic began, how often do you do any of the following?

|  | 1-Never | 2-Rarely | 3- Sometimes | 4-Often | 5-Always |
| --- | --- | --- | --- | --- | --- |
| Physical distancing (e.g., try to always stay 2 m away from others) |  |  |  |  |  |
| Masking in public |  |  |  |  |  |
| Staying home while mildly sick  (Note *mildly sick is defined as runny nose, mild cough, mild sore throat but no fever or trouble breathing)* |  |  |  |  |  |
| Downloading/Using the contact tracing app |  |  |  |  |  |
| Going to bars, pubs, nightclubs, lounges |  |  |  |  |  |

How beneficial do you think the following actions are in helping reduce the spread of COVID-19?

|  | 1-No benefit | 2-Little Benefit | 3- Moderate Benefit | 4-High benefit |
| --- | --- | --- | --- | --- |
| Physical distancing (e.g., try to always stay 2 m away from others) |  |  |  |  |
| Masking in public |  |  |  |  |
| Staying home while mildly sick |  |  |  |  |
| Downloading/Using the contact tracing app |  |  |  |  |
| Avoiding bars, pubs, nightclubs or lounges |  |  |  |  |

How important do you think the following actions are to preventing the spread of COVID-19? Rank the following actions from **most important to least important** with1 being ‘the most important’ and 5 being ‘the least important’.

|  | Ranking |
| --- | --- |
| Physical distancing (e.g., try to always stay 2 m away from others) |  |
| Masking in public |  |
| Staying home when mildly sick |  |
| Downloading/Using the contact tracing app |  |
| Avoiding bars, pubs, nightclubs or lounges |  |

How difficult do you think the following COVID-19 actions are to do? Rank the following COVID-19 actions from **least to most difficult** with 1 being ‘the least difficult’ and 5 being ‘the most difficult’.

|  | Ranking |
| --- | --- |
| Physical distancing (e.g., try to always stay 2 m away from others) |  |
| Masking in public |  |
| Staying home when mildly sick |  |
| Downloading/Using the contact tracing app |  |
| Avoiding bars, pubs, nightclubs or lounges |  |

For these actions, how much are you influenced by what people around you (e.g., your family, friends, coworkers) do? Example: if the people around you all physically distance, are you more likely to do so yourself?

|  | 1-Not influenced at all | 2-Slightly influenced | 3- Moderately influenced | 4-Very influenced |
| --- | --- | --- | --- | --- |
| Physical distancing (e.g., try to always stay 2 m away from others) |  |  |  |  |
| Masking in public |  |  |  |  |
| Staying home while mildly sick |  |  |  |  |
| Downloading/Using the contact tracing app |  |  |  |  |
| Avoiding bars, pubs, nightclubs or lounges |  |  |  |  |

- **If a business required masks to enter, would you be**:
  - Less likely to enter the business
  - More likely to enter the business
  - This would not affect my decision to enter the business
- **If you could easily discover which businesses required masks or the contact tracing app, would you be more likely to go there?** [Y/N]
- **Would you wear a mask if not wearing a mask resulted in a fine?**
  - I will wear a mask regardless of whether there is a fine or not
  - I will wear a mask if there is a $25 fine for not wearing a mask
  - I will wear a mask if there is a $100 fine for not wearing a mask
  - I will wear a mask if there is a $250 fine for not wearing a mask
  - I will not wear a mask under any circumstances

*The following questions give us information about your knowledge, beliefs and opinions around COVID-19.*

- **Do you know anyone who has been tested for COVID-19?** [Y/N]
- **Have you been tested for COVID-19?** [Y/N]
- **Do you know anyone who had/has COVID-19?** [Y/N]
- **Do you have any medical conditions that make you more susceptible to COVID-19? These include diabetes, high blood pressure, heart disease, lung disease, obesity, and any medication or conditions that weaken your immune system?** [Y/N]
- **What is your level of concern for getting COVID-19 yourself?** [Extremely concerned, very concerned, somewhat concerned, not concerned at all]
- **What is your level of concern for SPREADING COVID-19 to others around you?** [Extremely concerned, very concerned, somewhat concerned, not concerned at all]
- **Do you feel confident that you can keep you and your family safe from COVID-19?** [Y/N]
- **Would you receive a COVID-19 vaccine if it was available?** [Y/N]
- **Have you received an annual flu vaccine since 2018?** [Y/N]

*The questions below tell us about the platforms you use to get information.*

- **What, if any, social media platforms do you use?** (Check all that apply).
  - TikTok, Reddit, Pinterest, YouTube, Tumblr, Instagram, Facebook, Twitter, Snapchat, LinkedIn, Other, None
- **How do you get your news?** (Check all that apply).
  - Watch on the television, newspaper, internet news sources (NYT, Calgary Herald), social media (Twitter, Facebook), I do not watch/read the news, other.
- **Where do you get your health information about COVID-19? (Pick all that apply)**
  - Physician
  - Other heathcare provider (e.g., pharmacist, public health nurse)
  - Alberta Health or AHS website
  - Dr Deena Hinshaw’s Media Briefings
  - Friends and family
  - Twitter
  - Facebook
  - Instagram
  - Other social media platform (e.g., Reddit, TikTok)
  - Television (e.g., broadcast news like CBC, CTV, Global, etc.)
  - Radio
  - Print newspaper
  - Google and other internet searches
  - None
- **What are your most trusted sources of health information?** (Pick up to 5).
  - Physician
  - Other heathcare provider (e.g., pharmacist, public health nurse)
  - Alberta Health or AHS website
  - Dr Deena Hinshaw’s Media Briefings
  - Friends and family
  - Twitter
  - Facebook
  - Instagram
  - Other social media platform (e.g., Reddit, TikTok)
  - Television (e.g., broadcast news like CBC, CTV, Global, etc.)
  - Radio
  - Print newspaper
  - Google and other internet searches
  - None

*The questions below give us information on how you value risk. For each question, please choose the option you like the most. For example, if you prefer a 30% chance of receiving $40 (with a 70% chance of receiving $0) over receiving $20 for sure you should answer option B for questions 1.*

1. Option A: $20 for sure Option B: a 30% chance at $40, 70% chance receiving zero

2. Option A: $20 for sure Option B: a 40% chance at $40, 60% chance receiving zero

3. Option A: $20 for sure Option B: a 50% chance at $40, 50% chance receiving zero

4. Option A: $20 for sure Option B: a 60% chance at $40, 40% chance receiving zero

5. Option A: $20 for sure Option B: a 70% chance at $40, 30% chance receiving zero

*The questions below give us information on how you make trade-off over time. For each of the questions, please choose the option you like the most. For example, in question 1, if you prefer receiving $40 in two weeks over receiving $42 in six weeks, you should select option B as your answer.*

1. Option A: $40 in two weeks Option B: $42 in six weeks

2. Option A: $40 in two weeks Option B: $44 in six weeks

3. Option A: $40 in two weeks Option B: $46 in six weeks

4. Option A: $40 in two weeks Option B: $48 in six weeks

*The questions below are related to information about you.*

- **What is your occupation?** Employed for wages, self employed, Out of work, staying at home, a student, retired, unable to work, maternity or paternity leave
- **(SKIP if not employed for wages or self-employed above):** Since the pandemic began, have you mostly been going to work still or have you mostly been working from home?
- **What is the highest level of education you completed**: Grade school or some high school, High school diploma, Post-secondary technical school, Some college or university, College or University diploma/degree, Prefer not to answer
- **What are the first 3 digits of your postal code?**
- **Which ethnic group do you most strongly identify with?**
- White
- South Asian (e.g., East Indian, Pakistani, Sri Lankan, etc.)
- Chinese
- Black
- Filipino
- Latin American
- Arab
- Southeast Asian (e.g., Vietnamese, Cambodian, Malaysian, Laotian, etc.)
- West Asian (e.g., Iranian, Afghan, etc.)
- Korean
- Japanese

First Nations/ Metis/ Inuk/Inuit

- **Do you take public transit?** [Almost always, sometimes, rarely, never]
- **What is your marital** **status**? [married, living common law, never married, separated, divorced, widowed]
- **Do you have children under the age of 18 years living in your house?** [Y/N]
- **Do you make your own healthcare decisions?** [Y/N]
- **What is your living situation? Check all that apply.**
  - Alone,
  - With children
  - With parents,
  - With other family members
  - With non-family roommates
  - Other
  - Prefer not to answer.
- **Do you currently live in a house or an apartment?** House, Apartment
- **Do you own or rent your home?** Own, Rent
- **What is your annual household income?** <$10,000, $10,000 to less than $20,000, $20,000 to less than $30,000, ... ≥$100,000, prefer not to answer

**Supplementary Material: Quotas for recruitment parameters**- designed with the intent to pursue focus groups with the same population following the survey. The quotas are defined by age and location within Alberta.

- 2 Focus Groups: 18-29 year olds in Calgary
- 1 Focus Group: 18-29 year olds in Edmonton & other urban*
- 1 Focus Group: 30-59 year olds in Calgary
- 1 Focus Group: 30-59 year olds in Edmonton & other urban*
- 1 Focus Group: All ages in rural
- 1 Focus Group: >60 year olds in Calgary
- 1 Focus Group: >60 year olds in Edmonton & other urban*
- 1 Focus Group: All ages of parents with school-age children, no defined location.

*Other urban centers included: Red Deer, Medicine Hat, Lethbridge and Grand Prairie
